# HTRIdb: an open-access database for experimentally verified human transcriptional regulation interactions

**DOI:** 10.1186/1471-2164-13-405

**Published:** 2012-08-17

**Authors:** Luiz A Bovolenta, Marcio L Acencio, Ney Lemke

**Affiliations:** 1 Departamento de Física e Biofísica, Instituto de Biociências de Botucatu, Unesp - Univ Estadual Paulista, Distrito de Rubião Jr. s/n, Botucatu, São Paulo, 18618-970, Brazil

## Abstract

**Background:**

The modeling of interactions among transcription factors (TFs) and their respective target genes (TGs) into transcriptional regulatory networks is important for the complete understanding of regulation of biological processes. In the case of experimentally verified human TF-TG interactions, there is no database at present that explicitly provides such information even though many databases containing human TF-TG interaction data have been available. In an effort to provide researchers with a repository of experimentally verified human TF-TG interactions from which such interactions can be directly extracted, we present here the Human Transcriptional Regulation Interactions database (HTRIdb).

**Description:**

The HTRIdb is an open-access database that can be searched via a user-friendly web interface and the retrieved TF-TG interactions data and the associated protein-protein interactions can be downloaded or interactively visualized as a network through the web version of the popular Cytoscape visualization tool, the Cytoscape Web. Moreover, users can improve the database quality by uploading their own interactions and indicating inconsistencies in the data. So far, HTRIdb has been populated with 284 TFs that regulate 18302 genes, totaling 51871 TF-TG interactions. HTRIdb is freely available at http://www.lbbc.ibb.unesp.br/htri.

**Conclusions:**

HTRIdb is a powerful user-friendly tool from which human experimentally validated TF-TG interactions can be easily extracted and used to construct transcriptional regulation interaction networks enabling researchers to decipher the regulation of biological processes.

## Background

The modeling of interactions among transcription factors (TFs) and their respective target genes (TGs) into transcriptional regulatory networks is an important step for the complete understanding of regulation of biological processes since the ensemble of these interactions into a single interaction network model provides insight on the principles and properties that control differential gene expression at a systems level [1].

The first step for constructing a transcriptional regulatory network is gathering data from databases containing TF-TG interactions. The most prominent examples of such databases are JASPAR [2], the Open Regulatory Annotation database (ORegAnno; [3]), Swissregulon [4], TRANSFAC database [5], the Transcriptional Regulatory Element Database (TRED; [6]) and the Transcription Regulatory Regions Database (TRRD; [7]).

If one wants to construct, for example, a human transcriptional regulatory network containing only computationally predicted TF-TG interactions, one will easily infer these interactions from the above-mentioned databases since they all provide experimentally verified or computationally predicted human TF-DNA binding sites, specially JASPAR and Swissregulon that are mainly focused on TF-DNA binding sites. These binding sites can then be mapped to the entire genome and a TF-TG interaction is inferred if a binding site related to a certain TF is relatively close to the transcription start site of a certain gene.

On the other hand, if one wants to build a transcriptional regulatory network containing only experimentally verified human TF-TG interactions, i.e. interactions demonstrated by at least one experimental technique, one can try to extract these interactions from ORegAnno, TRANSFAC, TRED or TRRD. However, although these databases have undoubtedly been wealthy sources of transcriptional regulatory information for life scientists, some constraints limit their use to construct transcriptional regulatory networks. While TRANSFAC can not be freely accessed, TRRD and TRED does not have any mechanism for extracting their TF-TG interactions. ORegAnno, on the other hand, is freely accessible and provides TF-TG interactions in a form useful for constructing interaction networks; however, ORegAnno files containing TF-TG interactions should be parsed to remove non-human interactions and records with missing information.

In an effort to provide researchers with a repository of experimentally validated human TF-TG interactions from which such interactions can be easily obtained and directly used to construct transcriptional regulation networks, we describe here the Human Transcriptional Regulation Interactions database (HTRIdb), an open-access database of experimentally validated interactions among human TFs and their respective TGs, specifically physical interactions among TFs and their TGs promoters. HTRIdb can be searched via a user-friendly web interface (http://www.lbbc.ibb.unesp.br/htri) and the retrieved TF-TG interactions can be downloaded or visualized as a network through the embedded Cytoscape Web software [8]. Protein-protein interactions (PPIs) associated with the TF-TGs of interest can also be downloaded or visualized as a network.

## Construction and content

### Database design

The HTRIdb is implemented as a relational database PostgreSQL (http://www.postgresql.org) that is connected to a web interface via the JBOSS AS (http://www.jboss.org/jbossas/) that dynamically generates user-friendly HTML front-end queries using the Apache Tomcat web server (www.apache.org). For the network visualization of interactions, we embedded in HTRIdb the Cytoscape Web [8] according to instructions provided by the Cytoscape Consortium in http://cytoscapeweb.cytoscape.org/tutorial.

**Figure 1:**
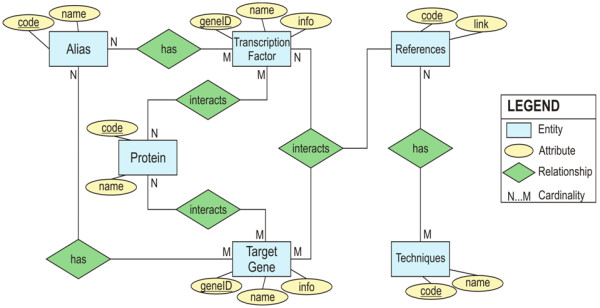
**Entity relationship diagram showing the schema for the HTRIdb.** The attribute “name” associated with the entities “Transcription Factor” , “Target Gene” and “Protein” represents the gene official symbols for, respectively, a TF, a TG and a TF/TG interacting protein. The attribute “name” associated with “Alias” represents the alias symbol for a TG or a TF. The attribute “name” associated with “Technique” represents a technique name. The attribute “info” represents the TFs’ and TGs’ links to their respective Entrez Gene report pages. The attribute “code” associated with entities “Alias”, “References” and “Techniques” represents an internal code specific for each entity; the attribute “code” associated with entity “Protein” represents the Entrez Gene ID of a TF/TG interacting protein.

The HTRIdb provides for each TF-TG interaction (1) the TF and TG gene official and alias symbols, (2) the Entrez Gene ID codes for both TF and TG, (3) links to the Entrez Gene report pages for both TF and TG, (4) the experimental technique used to detect the TF-TG interaction, (5) the Pubmed ID for the article reporting the interaction with a link to the respective article’s abstract page in Pubmed and (6) the protein-protein interactions (PPIs) of both TF and TG. Figure 1 shows the entity relationship diagram depicting the schema for the HTRIdb.

### Database content

At the time of writing, HTRIdb housed a collection of 51871 unique experimentally verified transcriptional regulation interactions among 284 TFs and 18302 TGs detected by 14 distinct techniques (these data are accessible via the “Statistics” button in the HTRIdb main page). Of these 51871 TF-TG interactions, 2283 were detected by small and mid-scale techniques (chromatin immunoprecipitation, concatenate chromatin immunoprecipitation, CpG chromatin immunoprecipitation, DNA affinity chromatography, DNA affinity precipitation assay, DNase I footprinting, electrophoretic mobility shift assay, southwestern blotting, streptavidin chromatin immunoprecipitation, surface plasmon resonance and yeast one-hybrid assay ) and 49588 interactions were detected by chromatin immunoprecipitation coupled with microarray (ChIP-chip) or chromatin immunoprecipitation coupled with deep sequencing (ChIP-seq).

Interactions detected by small and mid-scale techniques were collected from original research articles as follows. First, we performed a Pubmed search–limited to the title or abstract of English written journal articles focused on humans–using a Boolean complex query with the words “bind” and “interact” and some of their variants along with phrases containing several alternative names for the chromatin immunoprecipitation and electrophoretic mobility shift assay techniques (see the complete Boolean query in Additional file 1). This search strategy yielded 2471 articles (see the list of Pubmed IDs for these articles in the Additional file 1). We then manually checked each article for the presence of TF-TG interactions and associated detection techniques. Of these 2471 articles, we were able to extract the TF-TG interactions and associated small and mid-scale techniques from 893 articles. The remaining articles were discarded due to gene name ambiguity or lack of clear TF-TG interactions.

The checking for the presence of TF-TG interactions and associated detection techniques in articles was facilitated by an annotation tool developed by our group (available as a Mathematica notebook upon request) that highlights in the abstracts the gene names or symbols for TFs and TGs and the names for techniques. The gene names and symbols for TFs and TGs are taken from a list of gene official and alias names for genes (see Additional file 1) that we built from the *Homo sapiens* gene information file downloaded from the National Center for Biotechnology Information (NCBI) ftp site (ftp://ftp.ncbi.nih.gov/gene/). We considered as TFs those listed in the high-confidence data set of 1391 TFs produced by Vaquerizas and colleagues [9].

Interactions detected by ChIP-chip and ChIP-seq were also collected from original research articles, but in this case, we first selected ChIP-chip and ChIP-seq experiments from the hmChIp database [10] (see the list of Gene Expression Omnibus Series records for these experiments in Additional file 1), downloaded the corresponding articles and then extracted the interactions from the accompanying supplementary files. The PPIs of TFs and TGs, on the other hand, were extracted from a integrated network of human gene interactions recently published by our group [11].

**Figure 2:**
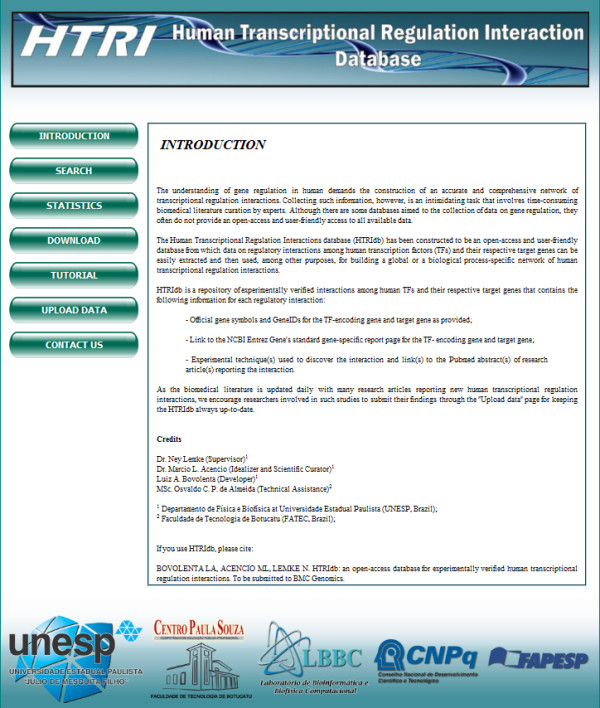
**Screenshot of the main page of HTRIdb.** Through the buttons in the left menu, users can access all HTRIdb features. To search TF-TG interactions data of interest, users should click the “SEARCH” button. To keep track of the number of TFs, TGs, TF-TG interactions, articles and techniques present in HTRIdb and verify the proportion of known human TFs covered by HTRIdb, users should click the“STATISTICS” button. To download all interactions–TF-TG or TF-TG with PPIs–users should click the “DOWNLOAD” button. To learn how to retrieve and download or visualize as a network the TF-TG interactions and PPIs of a given TF or TG of interest, users should click the “TUTORIAL” button. To add new TF-TG interaction data to HTRIdb by uploading their newly discovered TF-TG interactions data, users should click the “UPLOAD DATA” button. To send suggestions and comments or point out inconsistencies encountered in some TF-TG interaction to HTRIdb staff, users should click the “CONTACT US” button.

The 51871 TF-TG interactions currently present in HTRIdb is certainly much lower than the amount of existing TF-TG interactions in human. Therefore, to increase the number of TF-TG interactions in HTRIdb, we plan to update it periodically by manually curating the literature. Also, and more importantly: *researchers involved in transcriptional regulatory studies can contribute to HTRIdb by uploading their newly discovered TF-TG interactions data via the “Upload Data” page accessible from the main page of HTRIdb* (see Figure 2).

## Utility and discussion

### Database access and features

The HTRIdb is freely accessible via a user-friendly web interface at http://www.lbbc.ibb.unesp.br/htri. Besides searching for TF-TG interactions data of interest (see below), users can *(i)* download all interactions–TF-TG or TF-TG with PPIs–contained in the HTRIdb through the “Download” page, *(ii)* keep track of the number of TFs, TGs, TF-TG interactions, articles and techniques present in HTRIdb and verify the proportion of known human TFs covered by HTRIdb through the “Statistics” page, *(iii)* learn how to retrieve and download or visualize as a network the TF-TG interactions and PPIs of a given TF or TG of interest via the “Tutorial” page, *(iv)* add new TF-TG interaction data to HTRIdb, as previously mentioned, by uploading their newly discovered TF-TG interactions data via the “Upload Data” page, and *(v)* send suggestions and comments or point out inconsistencies encountered in some TF-TG interaction to HTRIdb staff via the “Contact Us” page (Figure 2).

**Figure 3:**
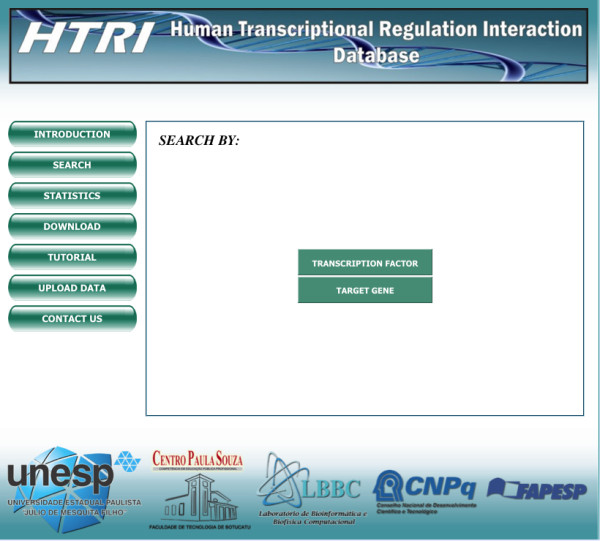
**Screenshot of the “Search” page.** In this page, users should click the “TRANSCRIPTION FACTOR” or the “TARGET GENE” buttons if they want to search TF-TG interactions data for, respectively, a TF or a TG of interest.

**Figure 4:**
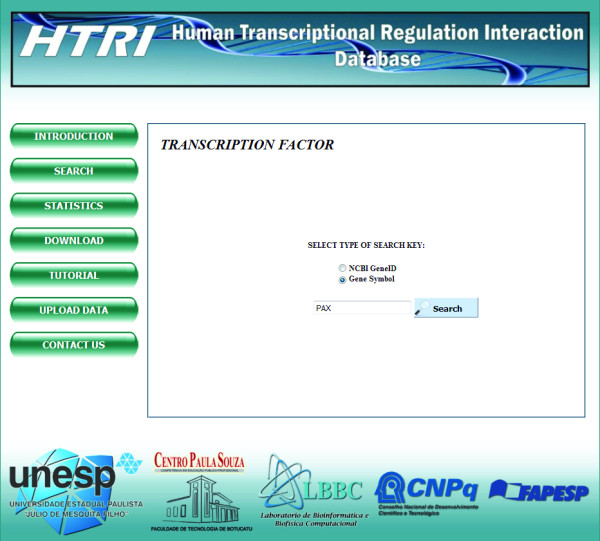
**Screenshot of the “Transcription Factor” page within “Search” page.** In this page, users should select the type of search key. If they know the TF’s EntrezGene ID, the “NCBI GeneID” option should be selected and the desired EntrezGene ID should be entered in the search field; otherwise, the “Gene Symbol” option should be selected and the complete or partial TF’s official gene or alias symbol should be entered in the search field. In this case, the user entered the TF’s gene partial symbol “PAX”.

**Figure 5:**
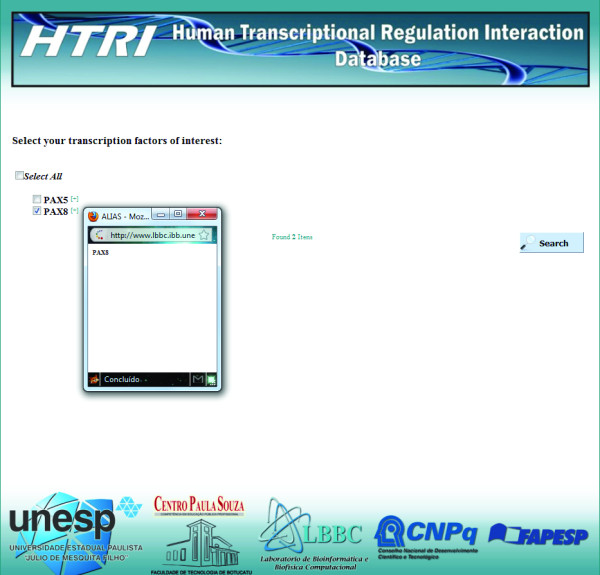
**Screenshot of the page to select and confirm the TF of interest.** This page shows a list of possible TF’s gene official symbols matching the entered query in the “Transcription Factor” page (in this case, “PAX”–see Figure 4). Here, *PAX8* was selected by the user as the TF of interest.

**Figure 6:**
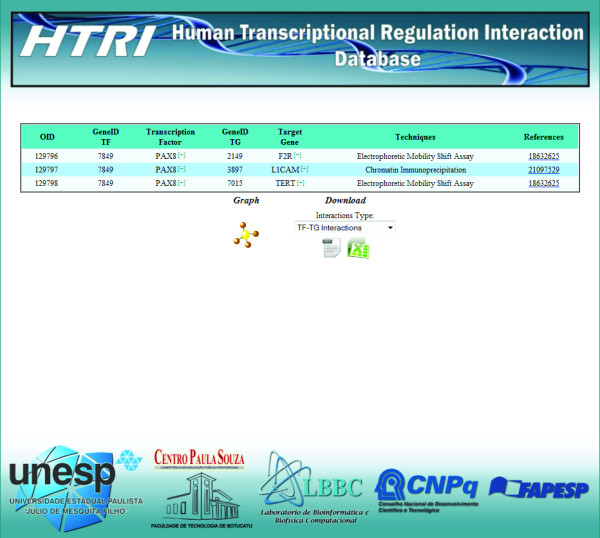
**Screenshot of the result page.** The result page displays all TF-TG interactions related to the TF of interest (in this case, *PAX8*). It contains TF’s NCBI GeneID, TF’s official gene symbol, link for additional information on TF in the Entrez Gene database, TG’s NCBI GeneID, TG’s official gene symbol, link for additional information on TG in the Entrez Gene database, the technique used to detect the TF-TG interaction and the link for the Pubmed abstract for the article describing the interaction. Moreover, by clicking the “Graph” or “Download” icons, users can, respectively, visualize the TF-TG interactions as a network or download TF-TG interactions data only or TF-TG interactions data associated with their PPIs in a tab-delimited or spreadsheet file.

Regarding the search mechanism (for a simple use case, please see the next section), users can retrieve TF-TG interactions data on a specific TF or TG of interest. For this purpose, users should access the “SEARCH” page and then proceed to the “TRANSCRIPTION FACTOR” or to the “TARGET GENE” page, depending on the type of search (Figure 3). In these pages, queries can be entered using the Entrez Gene ID code or the total or partial TF or TG gene official or alias symbols (Figure 4). This feature eliminates the need for users to know the exact name of TF or TG of interest; moreover, a page containing a list of possible TF or TG gene official symbols matching the entered query further helps users to confirm their choice (Figure 5). The retrieved data includes the Entrez Gene IDs, the gene official symbols and the links to the respective Entrez Gene report pages for both TFs and TGs and the name of experimental technique used to detect the interactions with the Pubmed IDs for articles reporting the discovery of these interactions (Figure 6). TF-TG interactions data only or TF-TG interactions data associated with their PPIs can be downloaded in a tab-delimited or spreadsheet file; moreover, TF-TG interactions can be visualized as a network by clicking the “Graph” icon (Figure 6).

**Figure 7:**
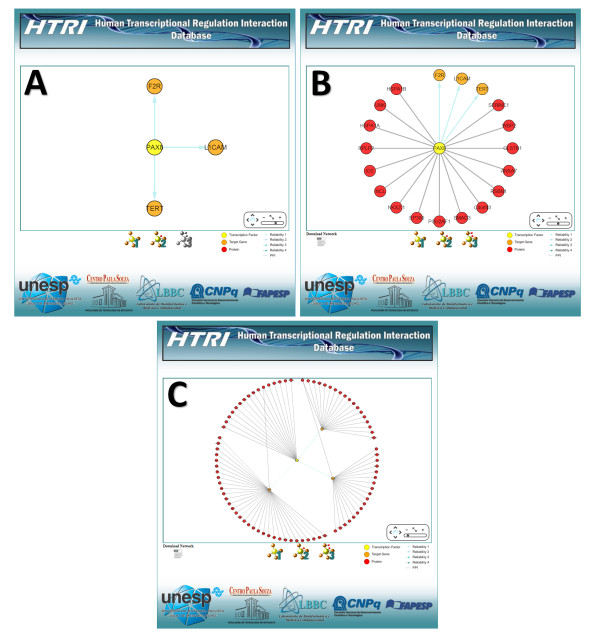
**Screenshots of the network visualization pages.** This tool is provided by the embedded Cytoscape Web software [5] and shows networks in three levels: first level displays only the interactions between the TF of interest and its TGs (**A**), second level displays interactions from first level plus PPIs between TF of interest and other proteins (**B**) and third level displays interaction from first and second level plus PPIs between TGs and other proteins (**C**).

In the network visualization tool provided by the embedded Cytoscape Web software [8], networks can be seen in three levels (Figure 7). The first level displays only the interactions between the TF of interest and its TGs (Figure 7a), the second level displays interactions from first level plus PPIs between TF of interest and other proteins (Figure 7b) and the third level displays interaction from first and second levels plus PPIs between TGs and other proteins (Figure 7c). The network displayed in the second and third levels can be downloaded in text format by clicking the“Download Network” icon (Figures 7b and 7c).

Through the network visualization of TF-TG interactions, users can also check the reliability of interactions. Reliability scores were assigned to interactions according to the number of articles reporting the interactions and the number of different techniques used to detect them (Table 1). These scores are coded with a color gradient from light blue (Reliability 1) to dark blue (Reliability 4) (Figure 7).

**Table 1:** Reliability scores for TF-TG interactions

**Score**	**Number of techniques**	**Number of articles**
Reliability 1	1	1
Reliability 2	1	> 1
Reliability 3	> 1	1
Reliability 4	> 1	> 1

Reliability scores depend on the number of articles reporting the interactions and the number of different techniques used to detect them.

### Case study: searching for *PAX8* target genes

In this section, we describe the steps for retrieving the target genes of TF *PAX8* and then visualizing the interactions between this TF and its TGs along with associated PPIs as a network. This case study serves both to reinforce key features of HTRIdb and as an example that could be followed to interrogate any TF or TG of interest. 

1. In the main page of HTRIdb, click the “SEARCH” button located in the left menu (Figure 2);

2. In the “SEARCH” page, select the“Transcription Factor” button (Figure 3);

3. In the “TRANSCRIPTION FACTOR” page, select the type of search key. If you know the TF’s EntrezGene ID, select the “NCBI GeneID” option and enter it in the search field; otherwise, select the “Gene Symbol” option and enter the complete or partial TF’s official gene or alias symbol. In this case, the TF of interest is *PAX8* and the user enters the TF’s gene partial symbol “PAX” (Figure 4);

4. After clicking the “Search” button in the “TRANSCRIPTION FACTOR” page, a page containing the list of possible gene official symbols matching “PAX” appears (Figure 5). In this page, select the official gene symbol that represents your TF of interest (in this case, PAX8) and then click the “Search” button. By clicking “[+]”, it is possible to see the selected TF’s alias symbols (Figure 5).

5. The result page displays all TF-TG interactions related to *PAX8* (Figure 6). To download TF-TG interactions data only or TF-TG interactions data associated with their PPIs, select the interactome type in the selection box in the “Download” area and then click the icons below the selection box to download the data in text format or in spreadsheet format (Figure 6);

6. To visualize the *PAX8* and its TGs along with associated PPIs as a network, click the “Graph” icon in the result page (Figure 6) and then navigate through the levels of the network visualization tool (Figure 7). The first level displays only the interactions between *PAX8* and its TGs (Figure 7a), the second level displays interactions from first level plus PPIs between *PAX8* and other proteins (Figure 7b ) and third level displays interaction from first and second levels plus PPIs between the *PAX8* TGs and other proteins (Figure 7c).

### Comparison to other related databases

As mentioned in the “Introduction”, the most prominent examples of TF-TG interactions databases are JASPAR [2], the Open Regulatory Annotation database (ORegAnno; [3]), Swissregulon [4], TRANSFAC database [5], the Transcriptional Regulatory Element Database (TRED; [6]) and the Transcription Regulatory Regions Database (TRDD; [7]). As JASPAR and Swissregulon are mainly focused on TF-DNA binding sites, we will compare our database only with those databases from which experimentally verified TF-TG interactions can be extracted, namely TRANSFAC, TRRD, TRED and ORegAnno.

Despite their remarkable usefulness as sources of experimentally verified human TF-TG interactions, TRANSFAC, TRED, TRRD and ORegAnno present some constraints that limit their use to construct human transcriptional regulatory networks in a feasible way in comparison to HTRIdb. We discuss below the advantages of HTRIdb over these databases.

Although TRANSFAC is considered the leading TF-TG interactions database, the advantage of HTRIdb over TRANSFAC is that HTRIdb is freely accessible. To take advantage of all HTRIdb features, including the downloading of all data present in HTRIdb, users are not required to make any subscription. On the other hand, as TRANSFAC is marketed as a commercial resource, users are required to make a paid subscription to access its contents and, accordingly, to download its TF-TG interactions.

**Figure 8:**
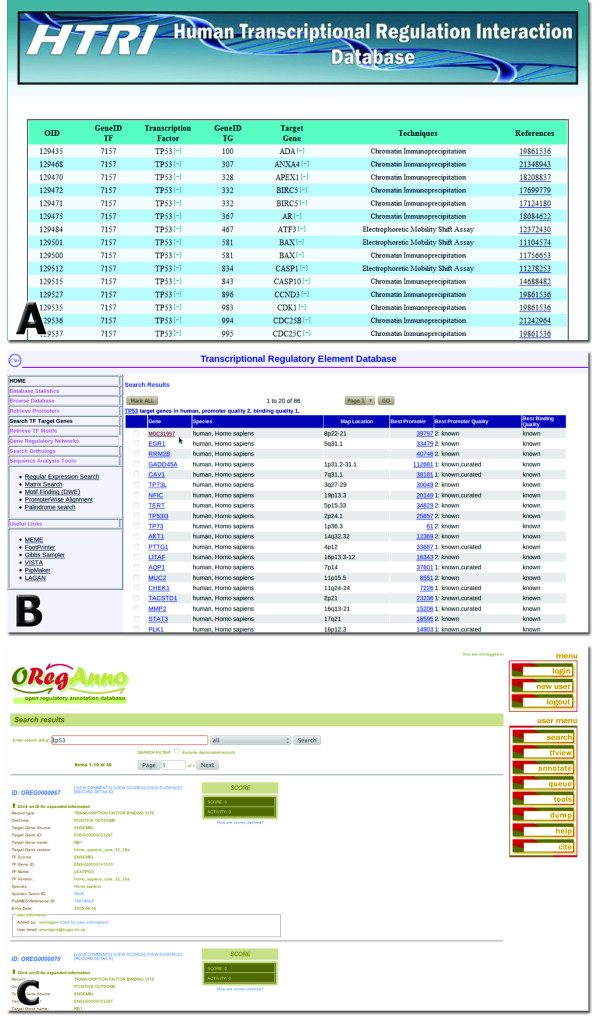
**Screenshots of the result pages of HTRIdb, TRED and ORegAnno.** The result page of HTRIdb displays all TF-TG interactions data in one page only (**A**), while the result pages of TRED (**B**) and ORegAnno (**C**) show partial TF-TG interactions data and are broken into subpages.

TRED and TRRD are freely accessible as HTRIdb is, but their major drawback is that they do not provide links to download their collection of TF-TG interactions. Conversely, HTRIdb, besides providing links to download the displayed results (Figure 6), also provides links to download all TF-TG interactions through the “DOWNLOAD” page that is easily accessible from the main page of HTRIdb (Figure 2). Besides the presence of download links, HTRIdb also offers additional advantages over TRED and TRRD, such as a dynamic network visualization tool, which is absent in TRRD and TRED (in fact, TRED provides only static images showing the interactions), and a more friendly result page. While the result page of TRED is broken into subpages containing a maximum of 20 TGs each in which techniques and Pubmed IDs are not displayed (Figure 8b), the result page of HTRIdb displays all TGs of the TF of interest along with the techniques used to detect the interactions and the Pubmed IDs for articles reporting the interactions (Figure 6 and Figure 8a). Regarding the result page of TRRD, links to pages showing further information on TF-TG interactions of interest are all displayed at once, but the names of links are non-obvious internal identifiers used by TRRD.

ORegAnno is also freely accessible as HTRIdb and TRED are and, differently from TRED and similarly to HTRIdb, provides links to download its entire collection of TF-TG interactions. The advantages of HTRIdb over ORegAnno mainly concern the following features: the format and quality of data contained in the downloaded file and the layout of the result page. With regard to format and data quality, ORegAnno tab-delimited flat files should be heavily parsed to remove non-human interactions, entries with missing information and gene name ambiguity; regarding the ORegAnno result page, it is broken into subpages containing a maximum of 10 TF-TG interactions each in which techniques used to detected the interactions are not displayed (Figure 8c). To find the technique, users should click the “RECORD DETAILS” link (Figure 8c). As already discussed above, the result page of HTRIdb displays all TGs of the TF of interest along with the techniques used to detect the interactions and the Pubmed IDs for articles reporting the interactions (Figure 6 and Figure 8a). In addition to the format and data quality of the downloaded flat file and layout of result page, HTRIdb also presents other two advantages over ORegAnno: (1) the network visualization tool and (2) a higher number of human TF and TG entries. While HTRIdb has 284 TFs and 18302 TGs, ORegAnno has 134 TFs and ∼ 1800 TGs.

## Conclusions

The development of HTRIdb is a response to an increasing need to centralize TF-TG interactions data in a user-friendly and open-access database from which investigators can easily extract data to construct either transcriptional regulation interactions or mixed protein-protein and transcriptional regulation interactions networks. As the construction of such networks is of utmost importance to decipher the regulation of biological processes at a systems level [1], we hope HTRIdb will become a useful tool for the systems biology research community.

## Availability and requirements

HTRIdb is available at http://www.lbbc.ibb.unesp.br/htri.

## Competing interests

The authors declare that they have no competing interests.

## Author’s contributions

LAB implemented the database and the front-end web interface and participated in the manual extraction of transcriptional regulation interactions. MLA conceived the idea of constructing the database, participated in the manual extraction of transcriptional regulation interactions and wrote the manuscript. NL directed the project. All authors read and approved the final manuscript.

## Supplementary Material

Additional file 1 Details about the search strategy used to collect the TF-TG interactions. This file includes (1) the Boolean query used in Pubmed search to retrieve articles that probably have human TF-TG interactions detected by small and mid-scale techniques, (2) the list of Pubmed IDs for articles retrieved after the search, (3) the list of gene official and alias symbols for human genes, (4) the list of gene official and alias names for human genes and (5) the list of Gene Expression Omnibus (GEO) Series records for the ChIP-chip experiments. Click here for file
